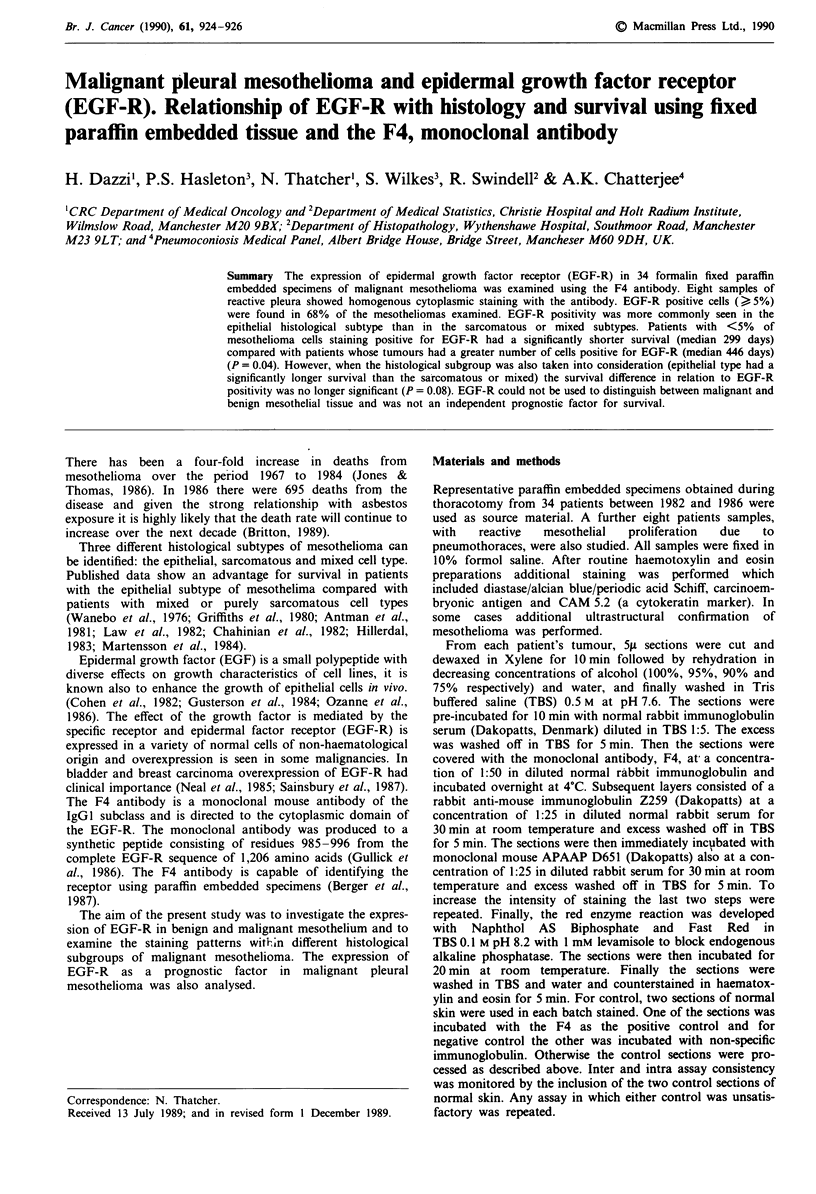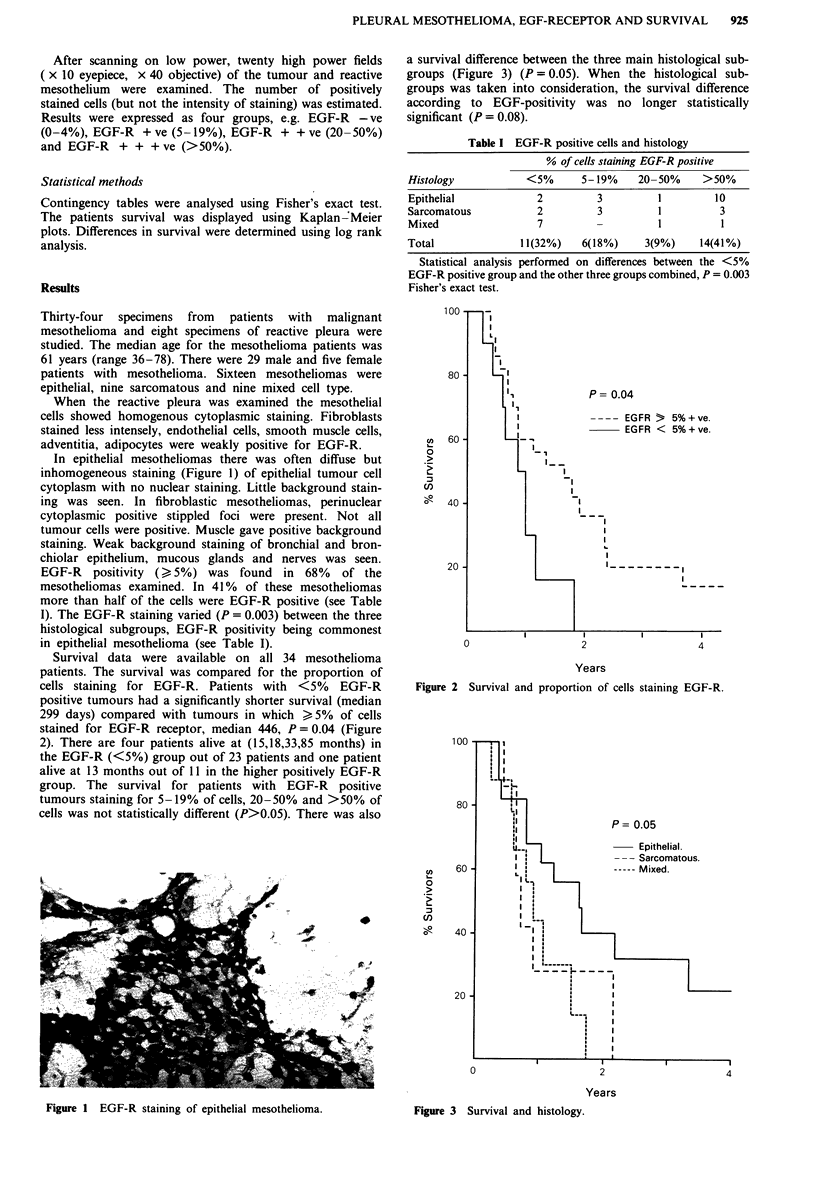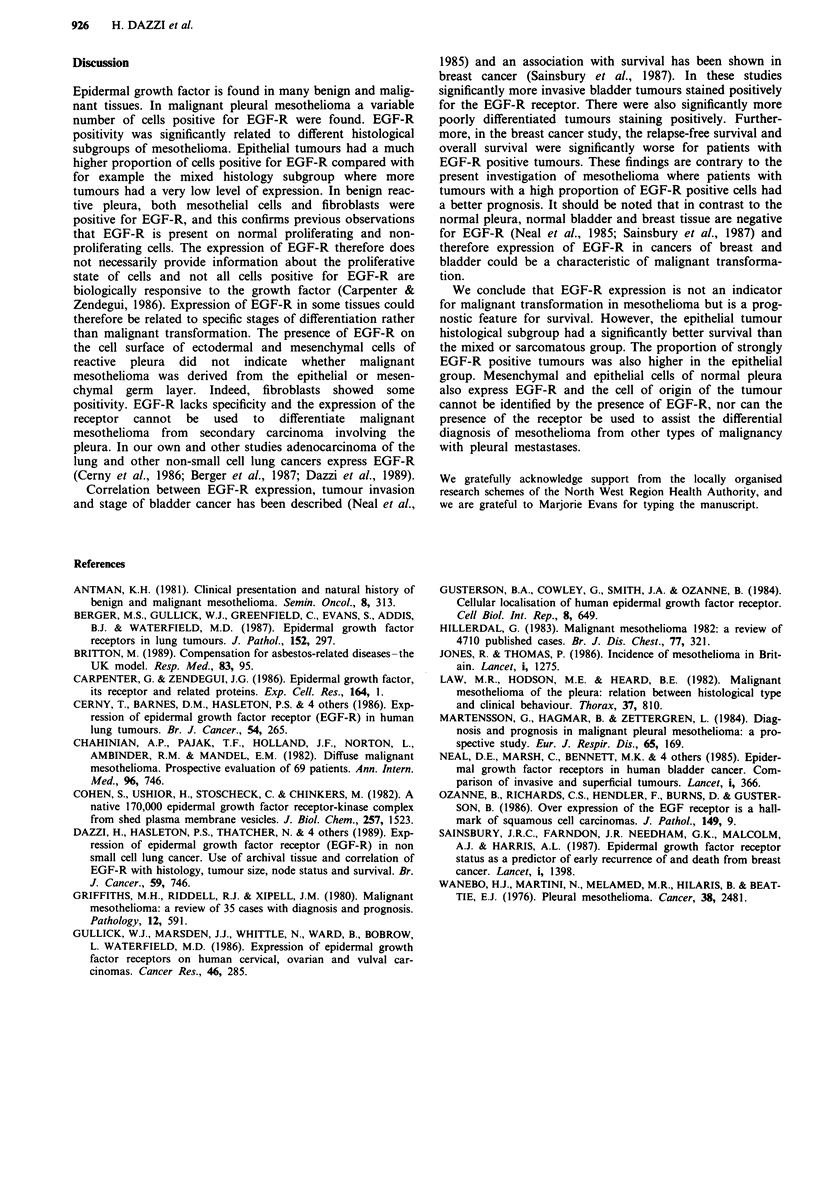# Malignant pleural mesothelioma and epidermal growth factor receptor (EGF-R). Relationship of EGF-R with histology and survival using fixed paraffin embedded tissue and the F4, monoclonal antibody.

**DOI:** 10.1038/bjc.1990.207

**Published:** 1990-06

**Authors:** H. Dazzi, P. S. Hasleton, N. Thatcher, S. Wilkes, R. Swindell, A. K. Chatterjee

**Affiliations:** CRC Department of Medical Oncology, Christie Hospital and Holt Radium Institute, Manchester, UK.

## Abstract

**Images:**


Br. J. Cancer (1990), 61, 924-926 © Macmillan Press Ltd., 1990

### Malignant pleural mesothelioma and epidermal growth factor receptor (EGF-R). Relationship of EGF-R with histology and survival using fixed paraffin embedded tissue and the F4, monoclonal antibody

H. Dazzi[1], P.S. Hasleton[3], N. Thatcher[1], S. Wilkes[3], R. Swindell[2] & A.K. Chatterjee[4]

[1]*CRC Department of Medical Oncology and* [2]*Department of Medical Statistics, Christie Hospital and Holt Radium Institute, Wilmslow Road, Manchester M20 9BX;* [2]*Department of Histopathology, Wythenshawe Hospital, Southmoor Road, Manchester M23 9LT; and* [4]*Pneumoconiosis Medical Panel, Albert Bridge House, Bridge Street, Mancheser M60 9DH, UK.*

**Summary** The expression of epidermal growth factor receptor (EGF-R) in 34 formalin fixed paraffin embedded specimens of malignant mesothelioma was examined using the F4 antibody. Eight samples of reactive pleura showed homogenous cytoplasmic staining with the antibody. EGF-R positive cells ($\geqslant 5\%$) were found in 68% of the mesotheliomas examined. EGF-R positivity was more commonly seen in the epithelial histological subtype than in the sarcomatous or mixed subtypes. Patients with $<5\%$ of mesothelioma cells staining positive for EGF-R had a significantly shorter survival (median 299 days) compared with patients whose tumours had a greater number of cells positive for EGF-R (median 446 days) ($P = 0.04$). However, when the histological subgroup was also taken into consideration (epithelial type had a significantly longer survival than the sarcomatous or mixed) the survival difference in relation to EGF-R positivity was no longer significant ($P = 0.08$). EGF-R could not be used to distinguish between malignant and benign mesothelial tissue and was not an independent prognostic factor for survival.

There has been a four-fold increase in deaths from mesothelioma over the period 1967 to 1984 (Jones & Thomas, 1986). In 1986 there were 695 deaths from the disease and given the strong relationship with asbestos exposure it is highly likely that the death rate will continue to increase over the next decade (Britton, 1989).

Three different histological subtypes of mesothelioma can be identified: the epithelial, sarcomatous and mixed cell type. Published data show an advantage for survival in patients with the epithelial subtype of mesothelima compared with patients with mixed or purely sarcomatous cell types (Wanebo *et al.*, 1976; Griffiths *et al.*, 1980; Antman *et al.*, 1981; Law *et al.*, 1982; Chahinian *et al.*, 1982; Hillerdal, 1983; Martensson *et al.*, 1984).

Epidermal growth factor (EGF) is a small polypeptide with diverse effects on growth characteristics of cell lines, it is known also to enhance the growth of epithelial cells *in vivo*. (Cohen *et al.*, 1982; Gusterson *et al.*, 1984; Ozanne *et al.*, 1986). The effect of the growth factor is mediated by the specific receptor and epidermal factor receptor (EGF-R) is expressed in a variety of normal cells of non-haematological origin and overexpression is seen in some malignancies. In bladder and breast carcinoma overexpression of EGF-R had clinical importance (Neal *et al.*, 1985; Sainsbury *et al.*, 1987). The F4 antibody is a monoclonal mouse antibody of the IgG1 subclass and is directed to the cytoplasmic domain of the EGF-R. The monoclonal antibody was produced to a synthetic peptide consisting of residues 985-996 from the complete EGF-R sequence of 1,206 amino acids (Gullick *et al.*, 1986). The F4 antibody is capable of identifying the receptor using paraffin embedded specimens (Berger *et al.*, 1987).

The aim of the present study was to investigate the expression of EGF-R in benign and malignant mesothelium and to examine the staining patterns within different histological subgroups of malignant mesothelioma. The expression of EGF-R as a prognostic factor in malignant pleural mesothelioma was also analysed.

Correspondence: N. Thatcher.
Received 13 July 1989; and in revised form 1 December 1989.

### Materials and methods

Representative paraffin embedded specimens obtained during thoracotomy from 34 patients between 1982 and 1986 were used as source material. A further eight patients samples, with reactive mesothelial proliferation due to pneumothoraces, were also studied. All samples were fixed in 10% formol saline. After routine haemotoxylin and eosin preparations additional staining was performed which included diastase/alcian blue/periodic acid Schiff, carcinoembryonic antigen and CAM 5.2 (a cytokeratin marker). In some cases additional ultrastructural confirmation of mesothelioma was performed.

From each patient's tumour, 5μ sections were cut and dewaxed in Xylene for 10 min followed by rehydration in decreasing concentrations of alcohol (100%, 95%, 90% and 75% respectively) and water, and finally washed in Tris buffered saline (TBS) 0.5 M at pH 7.6. The sections were pre-incubated for 10 min with normal rabbit immunoglobulin serum (Dakopatts, Denmark) diluted in TBS 1:5. The excess was washed off in TBS for 5 min. Then the sections were covered with the monoclonal antibody, F4, at a concentration of 1:50 in diluted normal rabbit immunoglobulin and incubated overnight at 4°C. Subsequent layers consisted of a rabbit anti-mouse immunoglobulin Z259 (Dakopatts) at a concentration of 1:25 in diluted normal rabbit serum for 30 min at room temperature and excess washed off in TBS for 5 min. The sections were then immediately incubated with monoclonal mouse APAAP D651 (Dakopatts) also at a concentration of 1:25 in diluted rabbit serum for 30 min at room temperature and excess washed off in TBS for 5 min. To increase the intensity of staining the last two steps were repeated. Finally, the red enzyme reaction was developed with Naphthol AS Biphosphate and Fast Red in TBS 0.1 M pH 8.2 with 1 mM levamisole to block endogenous alkaline phosphatase. The sections were then incubated for 20 min at room temperature. Finally the sections were washed in TBS and water and counterstained in haematoxylin and eosin for 5 min. For control, two sections of normal skin were used in each batch stained. One of the sections was incubated with the F4 as the positive control and for negative control the other was incubated with non-specific immunoglobulin. Otherwise the control sections were processed as described above. Inter and intra assay consistency was monitored by the inclusion of the two control sections of normal skin. Any assay in which either control was unsatisfactory was repeated.

After scanning on low power, twenty high power fields ( × 10 eyepiece, × 40 objective) of the tumour and reactive mesothelium were examined. The number of positively stained cells (but not the intensity of staining) was estimated. Results were expressed as four groups, e.g. EGF-R −ve (0–4%), EGF-R +ve (5–19%), EGF-R + +ve (20–50%) and EGF-R + + +ve (>50%).

*Statistical methods*

Contingency tables were analysed using Fisher's exact test. The patients survival was displayed using Kaplan-Meier plots. Differences in survival were determined using log rank analysis.

### Results

Thirty-four specimens from patients with malignant mesothelioma and eight specimens of reactive pleura were studied. The median age for the mesothelioma patients was 61 years (range 36–78). There were 29 male and five female patients with mesothelioma. Sixteen mesotheliomas were epithelial, nine sarcomatous and nine mixed cell type.

When the reactive pleura was examined the mesothelial cells showed homogenous cytoplasmic staining. Fibroblasts stained less intensely, endothelial cells, smooth muscle cells, adventitia, adipocytes were weakly positive for EGF-R.

In epithelial mesotheliomas there was often diffuse but inhomogeneous staining (Figure 1) of epithelial tumour cell cytoplasm with no nuclear staining. Little background staining was seen. In fibroblastic mesotheliomas, perinuclear cytoplasmic positive stippled foci were present. Not all tumour cells were positive. Muscle gave positive background staining. Weak background staining of bronchial and bronchiolar epithelium, mucous glands and nerves was seen. EGF-R positivity (⩾5%) was found in 68% of the mesotheliomas examined. In 41% of these mesotheliomas more than half of the cells were EGF-R positive (see Table I). The EGF-R staining varied ($P = 0.003$) between the three histological subgroups, EGF-R positivity being commonest in epithelial mesothelioma (see Table I).

Survival data were available on all 34 mesothelioma patients. The survival was compared for the proportion of cells staining for EGF-R. Patients with <5% EGF-R positive tumours had a significantly shorter survival (median 299 days) compared with tumours in which ⩾5% of cells stained for EGF-R receptor, median 446, $P = 0.04$ (Figure 2). There are four patients alive at (15,18,33,85 months) in the EGF-R (<5%) group out of 23 patients and one patient alive at 13 months out of 11 in the higher positively EGF-R group. The survival for patients with EGF-R positive tumours staining for 5–19% of cells, 20–50% and >50% of cells was not statistically different ($P > 0.05$). There was also a survival difference between the three main histological subgroups (Figure 3) ($P = 0.05$). When the histological subgroups was taken into consideration, the survival difference according to EGF-positivity was no longer statistically significant ($P = 0.08$).

**Figure 1** EGF-R staining of epithelial mesothelioma.

**Table I** EGF-R positive cells and histology

| | % of cells staining EGF-R positive | | | |
|---|---|---|---|---|
| *Histology* | <5% | 5–19% | 20–50% | >50% |
| Epithelial | 2 | 3 | 1 | 10 |
| Sarcomatous | 2 | 3 | 1 | 3 |
| Mixed | 7 | – | 1 | 1 |
| Total | 11(32%) | 6(18%) | 3(9%) | 14(41%) |

Statistical analysis performed on differences between the <5% EGF-R positive group and the other three groups combined, $P = 0.003$ Fisher's exact test.

**Figure 2** Survival and proportion of cells staining EGF-R.

**Figure 3** Survival and histology.

### Discussion

Epidermal growth factor is found in many benign and malignant tissues. In malignant pleural mesothelioma a variable number of cells positive for EGF-R were found. EGF-R positivity was significantly related to different histological subgroups of mesothelioma. Epithelial tumours had a much higher proportion of cells positive for EGF-R compared with for example the mixed histology subgroup where more tumours had a very low level of expression. In benign reactive pleura, both mesothelial cells and fibroblasts were positive for EGF-R, and this confirms previous observations that EGF-R is present on normal proliferating and non-proliferating cells. The expression of EGF-R therefore does not necessarily provide information about the proliferative state of cells and not all cells positive for EGF-R are biologically responsive to the growth factor (Carpenter & Zendegui, 1986). Expression of EGF-R in some tissues could therefore be related to specific stages of differentiation rather than malignant transformation. The presence of EGF-R on the cell surface of ectodermal and mesenchymal cells of reactive pleura did not indicate whether malignant mesothelioma was derived from the epithelial or mesenchymal germ layer. Indeed, fibroblasts showed some positivity. EGF-R lacks specificity and the expression of the receptor cannot be used to differentiate malignant mesothelioma from secondary carcinoma involving the pleura. In our own and other studies adenocarcinoma of the lung and other non-small cell lung cancers express EGF-R (Cerny *et al.*, 1986; Berger *et al.*, 1987; Dazzi *et al.*, 1989).

Correlation between EGF-R expression, tumour invasion and stage of bladder cancer has been described (Neal *et al.*, 1985) and an association with survival has been shown in breast cancer (Sainsbury *et al.*, 1987). In these studies significantly more invasive bladder tumours stained positively for the EGF-R receptor. There were also significantly more poorly differentiated tumours staining positively. Furthermore, in the breast cancer study, the relapse-free survival and overall survival were significantly worse for patients with EGF-R positive tumours. These findings are contrary to the present investigation of mesothelioma where patients with tumours with a high proportion of EGF-R positive cells had a better prognosis. It should be noted that in contrast to the normal pleura, normal bladder and breast tissue are negative for EGF-R (Neal *et al.*, 1985; Sainsbury *et al.*, 1987) and therefore expression of EGF-R in cancers of breast and bladder could be a characteristic of malignant transformation.

We conclude that EGF-R expression is not an indicator for malignant transformation in mesothelioma but is a prognostic feature for survival. However, the epithelial tumour histological subgroup had a significantly better survival than the mixed or sarcomatous group. The proportion of strongly EGF-R positive tumours was also higher in the epithelial group. Mesenchymal and epithelial cells of normal pleura also express EGF-R and the cell of origin of the tumour cannot be identified by the presence of EGF-R, nor can the presence of the receptor be used to assist the differential diagnosis of mesothelioma from other types of malignancy with pleural mestastases.

We gratefully acknowledge support from the locally organised research schemes of the North West Region Health Authority, and we are grateful to Marjorie Evans for typing the manuscript.

### References

ANTMAN, K.H. (1981). Clinical presentation and natural history of benign and malignant mesothelioma. *Semin. Oncol.*, **8**, 313.

BERGER, M.S., GULLICK, W.J., GREENFIELD, C., EVANS, S., ADDIS, B.J. & WATERFIELD, M.D. (1987). Epidermal growth factor receptors in lung tumours. *J. Pathol.*, **152**, 297.

BRITTON, M. (1989). Compensation for asbestos-related diseases – the UK model. *Resp. Med.*, **83**, 95.

CARPENTER, G. & ZENDEGUI, J.G. (1986). Epidermal growth factor, its receptor and related proteins. *Exp. Cell. Res.*, **164**, 1.

CERNY, T., BARNES, D.M., HASLETON, P.S. & 4 others (1986). Expression of epidermal growth factor receptor (EGF-R) in human lung tumours. *Br. J. Cancer.*, **54**, 265.

CHAHINIAN, A.P., PAJAK, T.F., HOLLAND, J.F., NORTON, L., AMBINDER, R.M. & MANDEL, E.M. (1982). Diffuse malignant mesothelioma. Prospective evaluation of 69 patients. *Ann. Intern. Med.*, **96**, 746.

COHEN, S., USHIOR, H., STOSCHECK, C. & CHINKERS, M. (1982). A native 170,000 epidermal growth factor receptor-kinase complex from shed plasma membrane vesicles. *J. Biol. Chem.*, **257**, 1523.

DAZZI, H., HASLETON, P.S., THATCHER, N. & 4 others (1989). Expression of epidermal growth factor receptor (EGF-R) in non small cell lung cancer. Use of archival tissue and correlation of EGF-R with histology, tumour size, node status and survival. *Br. J. Cancer.*, **59**, 746.

GRIFFITHS, M.H., RIDDELL, R.J. & XIPELL, J.M. (1980). Malignant mesothelioma: a review of 35 cases with diagnosis and prognosis. *Pathology*, **12**, 591.

GULLICK, W.J., MARSDEN, J.J., WHITTLE, N., WARD, B., BOBROW, L. WATERFIELD, M.D. (1986). Expression of epidermal growth factor receptors on human cervical, ovarian and vulval carcinomas. *Cancer Res.*, **46**, 285.

GUSTERSON, B.A., COWLEY, G., SMITH, J.A. & OZANNE, B. (1984). Cellular localisation of human epidermal growth factor receptor. *Cell Biol. Int. Rep.*, **8**, 649.

HILLERDAL, G. (1983). Malignant mesothelioma 1982: a review of 4710 published cases. *Br. J. Dis. Chest.*, **77**, 321.

JONES, R. & THOMAS, P. (1986). Incidence of mesothelioma in Britain. *Lancet*, **i**, 1275.

LAW, M.R., HODSON, M.E. & HEARD, B.E. (1982). Malignant mesothelioma of the pleura: relation between histological type and clinical behaviour. *Thorax*, **37**, 810.

MARTENSSON, G., HAGMAR, B. & ZETTERGREN, L. (1984). Diagnosis and prognosis in malignant pleural mesothelioma: a prospective study. *Eur. J. Respir. Dis.*, **65**, 169.

NEAL, D.E., MARSH, C., BENNETT, M.K. & 4 others (1985). Epidermal growth factor receptors in human bladder cancer. Comparison of invasive and superficial tumours. *Lancet*, **i**, 366.

OZANNE, B., RICHARDS, C.S., HENDLER, F., BURNS, D. & GUSTERSON, B. (1986). Over expression of the EGF receptor is a hallmark of squamous cell carcinomas. *J. Pathol.*, **149**, 9.

SAINSBURY, J.R.C., FARNDON, J.R. NEEDHAM, G.K., MALCOLM, A.J. & HARRIS, A.L. (1987). Epidermal growth factor receptor status as a predictor of early recurrence of and death from breast cancer. *Lancet*, **i**, 1398.

WANEBO, H.J., MARTINI, N., MELAMED, M.R., HILARIS, B. & BEATTIE, E.J. (1976). Pleural mesothelioma. *Cancer*, **38**, 2481.